# Deletion of the *GntR8* transcriptional regulator impairs *Brucella abortus* intracellular survival and virulence by modulating stress response genes

**DOI:** 10.3389/fimmu.2025.1698057

**Published:** 2025-10-29

**Authors:** Shuwen Li, Kun Han, Xiaowei Peng, Nan Wang, Wenqing Ning, Shengxin Ge, Lei Xu, Jiabo Ding, Xinyu Zhang, Xiaowen Yang

**Affiliations:** ^1^ Key Laboratory of Animal Biosafe Risk Prevention and Control (North), Ministry of Agriculture and Rural Affairs, Institute of Animal Science, Chinese Academy of Agricultural Sciences, Beijing, China; ^2^ College of Veterinary Medicine, Yangzhou University, Yangzhou, China; ^3^ College of Veterinary Medicine, Northwest A&F University, Xianyang, China; ^4^ National Reference Laboratory for Animal Brucellosis, China Institute of Veterinary Drug Control, Beijing, China; ^5^ College of Veterinary Medicine, Shandong Agricultural University, Taian, China

**Keywords:** *Brucella abortus*, GntR transcription factors, *clpP* gene, virulence, oxidative stress

## Abstract

GntR transcription factors are emerging as critical regulators of bacterial metabolism, stress responses, and pathogenicity, however, their roles in the virulence mechanisms of *Brucella abortus* remain poorly understood. In this study, we generated a *gntR8* (BAB_RS24500) deletion strain (Δ*gntR8*) in *B. abortus* 2308 and systematically investigated its role in virulence. The results demonstrate that deletion of *gntR8* markedly impairs intracellular survival of *B. abortus* in RAW264.7 cells and significantly reduces virulence in a mouse infection model. Moreover, the Δ*gntR8* strain exhibited increased sensitivity to oxidative stress, correlating with decreased expression of stress response genes. Integrative Dap-seq and RNA-seq analyses revealed that GntR8 directly binds to and positively regulates the *clpP* gene, a key component involved in oxidative stress defense. Deletion of *clpP* similarly resulted in diminished antioxidant capacity and intracellular survival, supporting a critical regulatory axis mediated by GntR8. Collectively, these findings provide novel insights into the molecular mechanisms by which GntR8 transcriptionally regulates oxidative stress responses and pathogenicity in *B. abortus*. The identification of GntR8 as a key virulence regulator highlights its potential as a therapeutic target, offering promising avenues for novel intervention strategies against brucellosis.

## 1 Introduction

Brucellosis is a zoonotic infectious disease caused by *Brucella* spp., which poses a significant threat to the health of both humans and animals (1). Humans typically acquire infection through direct contact with infected animals or ingestion of contaminated food products (2). Clinically, human brucellosis manifests as fever, malaise, arthralgia, sweating, and enlargement of the liver, spleen, and lymph nodes (3, 4). In livestock, *Brucella* infection primarily causes reproductive disorders such as abortion in females (5), orchitis and infertility in males, leading to substantial economic losses in livestock industry (6).

The pathogenicity of *Brucella* spp. is largely attributed to its ability to replicate and survive within host cells, thereby evading host immune responses (7). Transcription factors play crucial roles in regulating *Brucella* virulence and metabolic processes (8). For instance,

The BvrRS transcription factor, also known as a two-component regulator, controls the expression of genes required for multiple stages of *Brucella* infection. Studies have shown that Δ*bvrR* and Δ*bvrS* mutant strains are highly attenuated (9–11). BvrRS directly regulates the transcription of *Brucella* outer membrane protein genes *omp25*, *omp22*, and genes involved in lipopolysaccharide (LPS) modification (12, 13). It indirectly activates T4SS and outer membrane protein-related gene expression by inducing the expression of the gene encoding the quorum sensing regulator VjbR (14). The transcription regulator CtrA is the primary regulator of the *Brucella* cell cycle. Its activity is modulated by the histidine kinase PdhS, the CckA-ChpT phosphorylation cascade, and the protease adapter CpdR in response to endogenous cell cycle signals (15–18). The zinc finger protein MucR functions as a global regulator playing a crucial role in *Brucella* virulence (19). In *B. melitensis*, MucR influences LPS and correlates with oxidative stress tolerance. The MucR protein inhibits its own transcription and affects flagellar gene expression via the ftcR gene (20). In *B. abortus* 2308, MucR regulates genes associated with cell-membrane integrity, polysaccharide synthesis, and iron homeostasis (21).

The GntR family transcription factor represents another key regulatory system involved in bacterial metabolism and virulence. First described in 1987 (22), this family is subdivided into subfamilies such as MocR, YtrR, FadR, AraR, HutC, PlmA, DevA, and DasR, based on differences in their C-terminal amino acid sequences (23, 24). Previous studies have shown that deletion of the *gntR10* gene significantly affects *Brucella* growth and virulence in mice, modulates the expression of LuxR-type transcriptional activators (VjbR and BlxR), and influences the expression of type IV secretion system (T4SS) effectors (BspE and BspF) (25, 26). The reference strain *B. melitensis* 16M encodes 21 GntR transcription factors, of which seven have been implicated in virulence regulation (27, 28). Among them, GntR4(coded by BMEI0169), GntR12(coded by BMEII0807) and GntR17(coded by BMEI0320) are known to regulate the expression of the *virB* gene in *Brucella* spp (8, 27, 29)., and GntR17 additionally influences the expression of *omp25*, *vjbR* and *babR* genes in *B. abortus (* 8, 29). Previous study has revealed that the transcription factor GntR10 (BAB_RS31770) from B. abortus 2308 interacts with the target promoter of BAB1_1163 through sequence-specific DNA recognition, regulating the expression of 88 genes, including those involved in molecular functions, biological processes (BPs), and cellular components (CCs). The GntR10 target-gene mutant BAB1_1163 exhibits reduced expression in RAW 264.7 cells, affecting pro-inflammatory cytokine expression levels (26).

Despite these advances, relatively little is known about the broader role of other GntR transcription factors in virulence regulation of *Brucella*. Therefore, in this study, we constructed GntR transcription factor deletion and complemented strains using homologous recombination techniques, with *B. abortus* 2308 as the parental strain. We systematically examined the impact of *gntR8* (BAB_RS24500) deletion on virulence at the bacterial, cellular, and animal levels. Additionally, using DNA affinity purification sequencing (Dap-seq) and Electrophoretic mobility shift assay (EMSA), we identified and verified downstream target genes regulated by *gntR8* gene and performed functional analyses of these targets. This comprehensive analysis expands our understanding of GntR-mediated transcriptional regulation in *Brucella* and highlights GntR8 as a promising therapeutic target to disrupt virulence pathways. Targeting GntR8-mediated regulation could provide new approaches for reducing the economic and health impacts associated with brucellosis, suggesting future research should focus on therapeutic interventions designed to impair these transcriptional networks and mitigate disease transmission.

## 2 Materials and methods

### 2.1 Bacterial strains and cells

All Brucella strains were cultured in Tryptic Soy Agar (TSA, BD) or Tryptic Soy Broth (TSB, BD) at 37°C with 5% CO_2_. All work involving Brucella spp. strains was conducted in a biosafety Level 3 laboratory of China Institute of Veterinary Drug Control. The cells used in the in vitro experiments of this study were the RAW264.7 cells. The cell-culture conditions were DMEM medium (Gibco, USA) containing 10% fetal bovine serum, 100 U/mL penicillin, and 100 μg/mL streptomycin, cultured in a 37°C, 5% CO_2_ incubator.

### 2.2 Construction of *gntR8* deletion and complementation strains

The *gntR8* mutant strain was constructed following a previously published protocol (30). Primer sequences for deletion and complementation strains are listed in **Supplementary Table S1**. PCR products were cloned using the ClonExpress MultiS One Step Cloning Kit (Vazyme, China) and transformed into *E. coli* DH5α competent cells (CWbio, China). Positive plasmids were then electroporated into *Brucella* strains. Ampicillin-sensitive and chloramphenicol-sensitive colonies were verified by PCR to ensure successful genes deletion and complementation.

### 2.3 Cell infection assay

Intracellular survival assays of wild-type (WT) *B. abortus* 2308 and its mutant strains was performed as previously described (31). Briefly, RAW264.7 cells (2.5×10^5^ cells/well) were cultured in 24-well plates (Corning, USA) and infected with Brucella strains (100 MOI). Cells were then centrifuged at 1000 rpm for 10 minutes. After 1 h incubation, cells were washed three times with phosphate-buffered saline (PBS) and cultured in medium containing 50 μg/mL gentamicin. Wash twice more with PBS, then add 1 mL of medium containing 25 μg/mL gentamicin to each well. At 1, 24, and 48 hours post-infection (hpi), cells were lysed, and intracellular bacterial counts were determined by plating serial dilutions onto TSA plates.

### 2.4 Mice infection experiments

To evaluate the pathogenicity of gntR8 mutants in vivo, 80 female BALB/c mice (6–8 weeks) were randomly divided into four groups: PBS group, WT group, *ΔgntR8* group, and complemented strain (*CΔgntR8*) group (n=20). Each group was further subdivided into four time points, with five mice per group at each time point (n=5). Each mouse in the infection group received an intraperitoneal injection of 1×10^5^ CFU/0.1 mL of bacterial solution diluted in PBS, while the PBS group received an injection of 0.1 mL of PBS. Blood samples were collected from mice at weeks 1, 2, 3, and 4 post-infection. Mice were euthanized by asphyxiation at weeks 1, 2, 3 and 4 post-infection. After collection, spleen was weighed and divided into three portions. One portion was homogenized in 1 mL of PBS and subsequently subjected to TSA plate culture to determine bacterial load (n=5). Another portion was reserved for histopathological evaluation, and the third portion was used for RNA-seq analysis.

### 2.5 Histopathological evaluation

Histological examination was performed on spleen tissues as previously described (31). Briefly, spleen tissues were fixed in 10% formalin solution, embedded in paraffin, and sectioned into 4 µm slices using a microtome (Leica, Germany). Sections were stained with hematoxylin and eosin (HE) and examined under a light microscope (Leica, Germany).

### 2.6 Stress assays

Bacterial sensitivity under oxidative and acidic stress conditions was evaluated using a modified protocol (31–33).

Oxidative stress. WT, Δ*gntR8* and CΔ*gntR8* strains were treated with H_2_O_2_ at final concentrations of 1 mM, 2.5 mM and 5 mM, respectively. After 1 h of treatment at 37°C, surviving bacteria were enumerated by plating serial dilutions on TSA.

Acid stress. Bacterial cultures were pelleted by centrifugation, resuspended in TSB adjusted to pH 4.5 or 5.5, and incubated at 37°C for 1 h. Surviving bacteria were quantified by plating serial dilutions onto TSA plates.

### 2.7 GSSG and GSH assay

Intracellular concentrations of reduced glutathione (GSH) and oxidized glutathione (GSSG) in bacterial cells were determined using a GSH and GSSG Assay Kit (Beyotime, China), following the manufacturer’s instructions with minor modifications (34). A standard curve was established using the standards in the kit. Absorbance at 412 nm was measured after 25 min incubation at room temperature using a microplate reader. Intracellular GSH was calculated using the equation: GSH = total glutathione - GSSG × 2.

### 2.8 Quantitative real-time PCR analysis and RNA-sequencing

Total RNA from infected RAW264.7 cells and mice spleen tissues were isolated using TRIzol (Thermo Scientific, USA) according to the manufacturer’s instructions, followed by DNase I treatment to eliminate genomic DNA contamination. RNA concentration and purity were assessed using an ND 1000 spectrophotometer (Thermo Scientific, USA). Reverse transcription into cDNAs were synthesized using the PrimeScript RT Reagent Kit (TaKaRa Bio, Japan) according to the manufacturer’s instructions. Quantitative real-time PCR (qPCR) was performed with the primers shown in **Supplementary Table S2**. Relative gene expression was calculated using the comparative cycle threshold method (2^−ΔΔCt^), and each sample was analyzed in triplicate.

Sequencing libraries for each RNA sample were prepared using the NEB Next Ultra Directional RNA Library Prep Kit for Illumina according to the manufacturer’s protocol (35). RNA fragments were reverse-transcribed, amplified to double-stranded cDNA, adaptor- ligated, purified with magnetic bead and quantified. Sequencing was performed on the HiSeq 4000 platform at the Majorbio platform (Shanghai, China) with three biological replicates per group. Differential expression thresholds are fold change ≥ 2 and p ≤ 0.05.

### 2.9 Expression and purification of recombinant GntR8 protein

The coding region of the *gntR8* gene was amplified using primers pCold-G8-F and pCold-G8-R (**Supplementary Table S3**), digested with *Bam*H I and *Hind* III and cloned into the plasmid pCold II. The resulting plasmid (pCold-gntR8) was transformed into *E. coli* strain BL21. Protein expression was induced with 0.1 mM IPTG at 16 °C for 24 h. Cells were harvested, lysed by sonication, and centrifuged (12,000 × g, 20 min, 4 °C). The supernatant containing recombinant GntR8 protein was purified using Ni-Sepharose affinity chromatography.

### 2.10 DNA affinity purification sequencing

Purified GntR8 protein was flash-frozen into liquid nitrogen and stored at -80°C until use. Dap-seq analysis was performed by Yung Biotechnology Co., Ltd. (Beijing, China). Fastp (v0.20.1) software was used for quality control analysis of target proteins and negative controls, including removal of splices, repeats and low-quality sequences. Peak Calling was performed using MACS2 (v2.2.7.1) software (Fold change ≥ 2 and p-value ≤ 0.05) and the ChIPseeker (R package) was used to annotate peak. Motif analysis was conducted using HOMER software (v4.11.1).

### 2.11 Electrophoretic mobility shift assay

Biotin-labeled DNA probes were incubated in EMSA buffers (750 mM NaCl, 0.5 mM dithiothreitol (DTT), 0.5 mM EDTA, 50 mM Tris, pH 7.4) at 37°C for 30 min. For competitive assays, 100 nM unlabeled DNA probes were added to labeled probes. GntR8 protein (0–200 ng) was incubated with the probes at 37°C for 30 minutes. The samples were separated on a 6% Native-PAGE - gel (30% acrylamide, 5×TBE, TEMED, 10% APS, 5% Glycerol) and run in a 0.5 × Tris-Borate buffer (89mM Tris-Borate, 2mM EDTA, pH 7.4) at 200 V and 4 °C. Imaging was captured using a Typhoon FLA 9500 multifunctional scanner (GE Healthcare, USA).

### 2.12 Cytokine measurement by the multiplex cytokine assay system

Measure cytokine concentrations in mouse serum using the Luminex Flex MAP 3D system according to the manufacturer’s instructions (36). In brief, mix chemically labeled antibody-conjugated beads with standard solutions or samples, incubate overnight at 4°C, wash, and then incubate with biotinylated detection antibodies. After washing the beads, incubate them with streptavidin-phycoerythrin complexes. The sample is then washed using a handheld magnet and resuspended in sheath fluid. Finally, the sample is run on the Luminex FLEXMAP 3D® (Austin, Texas, USA), and data is collected and analyzed using MILLIPLEX Analyst 5.1 (Luminex). Three biological replicates were performed for each experimental group.

### 2.13 Statistical analysis

Basic statistical analyses were performed using GraphPad Prism 9.0 (USA). Unpaired Student’s t-tests were employed in cellular and mouse infection models, growth curve measurements of *gntR8* deletion strains, and bacterial virulence assays. For stress analyses, data were analyzed using analysis of variance (ANOVA). Data are expressed as mean ± standard deviation. *P* values < 0.05 were considered statistically significant.

## 3 Results

### 3.1 Deletion of *gntR8* significantly reduces *B. abortus* virulence in RAW264.7 cells and mice

**Figure 1:**
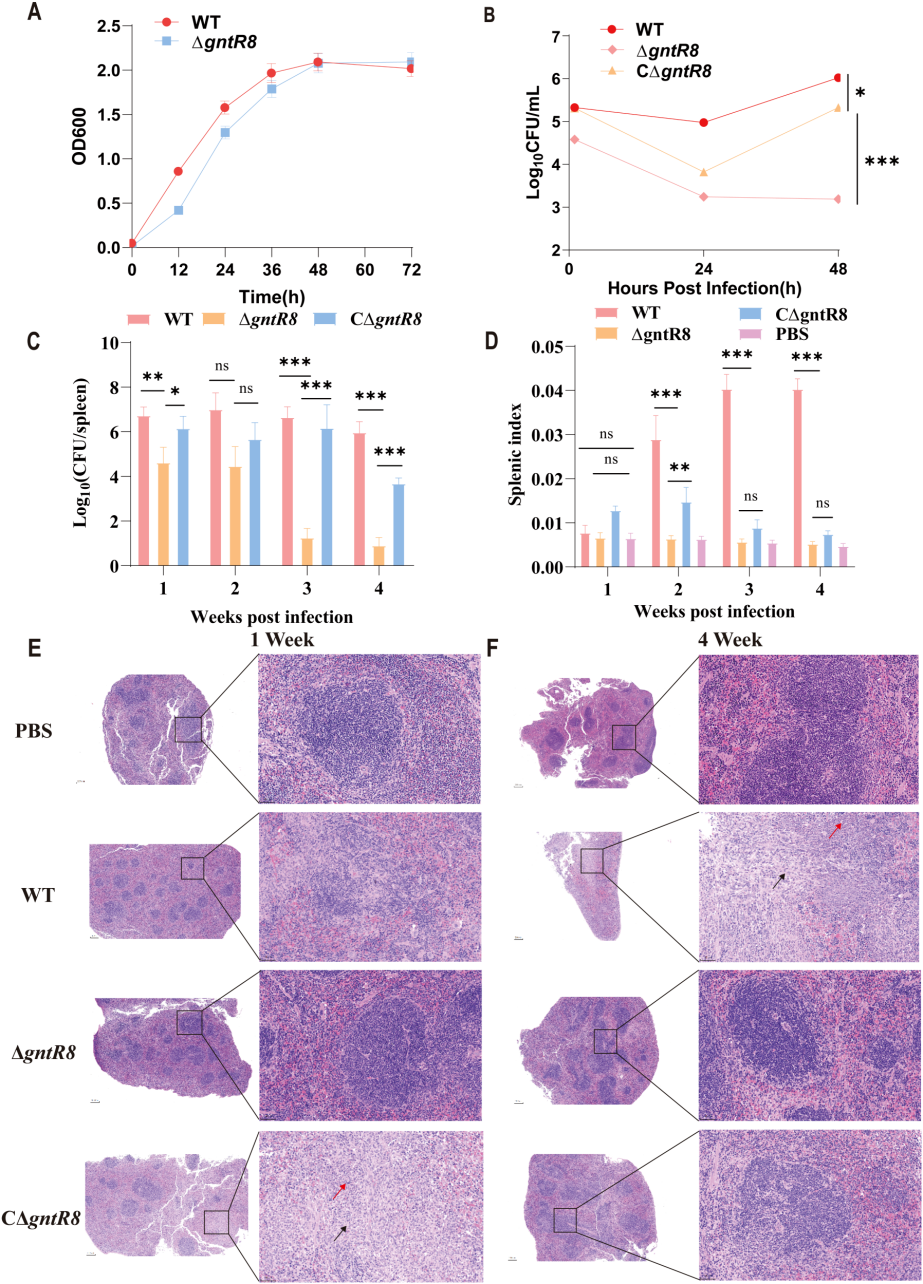
Deletion of the *gntR8* gene reduces **(*B*)** *abortus* survival within cells and its pathogenicity in mice. **(A)** Growth curves in TSB of *Brucella* strains at 37°C with continuous shaking for 72 (H) **(B)** Intracellular survival of RAW264.7 cells (100 MOI), values represent the means of three experiments performed in duplicate, and error bars indicate the SD; **(C)** Splenic bacterial load post-infection (n=5); **(D)** Splenic index post-infection (n=5); **(E-F)** Histopathological analysis of spleen at 1 week **(E)** and 4 week **(F)** post-infection with WT and *ΔgntR8*. Connective tissue hyperplasia is shown by red arrows, and inflammatory cell infiltration is shown by black arrows. Data are presented as the mean ± standard deviation (error bars) of standardized data, based on experimental results from five mice. The significance is shown as *p < 0.05; **p < 0.01; ***p < 0.001; and ns indicates non-significance.

Previous studies identified 21 GntR family transcription factors in the *B. melitensis* 16M strain (27). In this study, we analyzed the genome of *B. abortus* 2308 and identified 23 GntR family transcription factors through KEGG homologous gene analysis combined with NCBI database searches. As shown in **Supplementary Figure S1 A and B** and **Supplementary Table S4**, these factors include 7 located on chromosome I and 17 on chromosome II. To investigate the role of *gntR8*, we constructed a deletion strain (*ΔgntR8*) by replacing the *gntR8* gene with a kanamycin resistance gene via homologous recombination, and a complemented strain (C*ΔgntR8*) using the pBBRMCS-1 plasmid. Growth curves showed no significant difference between WT and *ΔgntR8* strain under normal culture conditions (**Figure 1A**). However, RAW264.7 cells infection assay indicated significant decrease in intracellular viability following infection with *ΔgntR8* strain compared to WT strain (*p* < 0.001) (**Figure 1B**). In addition, we quantified colocalization of intracellular *Brucella* with LAMP-1–positive compartments using laser confocal microscopy. At 4 h post-infection, the Δ*gntR8* mutant displayed a significantly higher LAMP-1 colocalization rates rather than the wild-type (WT) (*p* = 0.003) (**Supplementary Figure S2**), indicating impaired lysosomal evasion. To further investigate the effect of the *gntR8* gene deletion on *Brucella* virulence, we conducted mouse infection over 4 weeks to assess the survival of *ΔgntR8*, C*ΔgntR8*, and WT strains. As shown in **Figure 1C**, *ΔgntR8*-infected mice exhibited significantly lower bacterial loads in the spleen at 1, 3, and 4 weeks post-infection compared to the WT and C*ΔgntR8* groups (*p* < 0.001). Additionally, the spleen indices of WT-infected and C*ΔgntR8*-infected mice were significantly higher than those of the *ΔgntR8*-infected group at 2 weeks post-infection (*p* < 0.001) (**Figure 1D**). Histopathological analysis revealed no significant lesions in spleens at week 1 post-infection for both WT and Δ*gntR8* strains. However, spleens of the *ΔgntR8*-infected mice exhibited a marked reduction in lymphocytes and an increase in connective tissue proliferation (**Figure 1E**). At week 4 post-infection, spleens of mice infected with WT showed extensive connective tissue proliferation (red arrowheads) and focal neutrophil infiltration (black arrowheads). In contrast, no significant abnormalities were observed in the spleens *ΔgntR8*-infected groups (**Figure 1F**). These results indicate that the deletion of the *gntR8* gene significantly reduces the virulence of *B. abortus* both *in vivo* and *in vitro*.

### 3.2 Deletion of the *gntR8* reduces oxidative stress resistance in *B. abortus*

**Figure 2:**
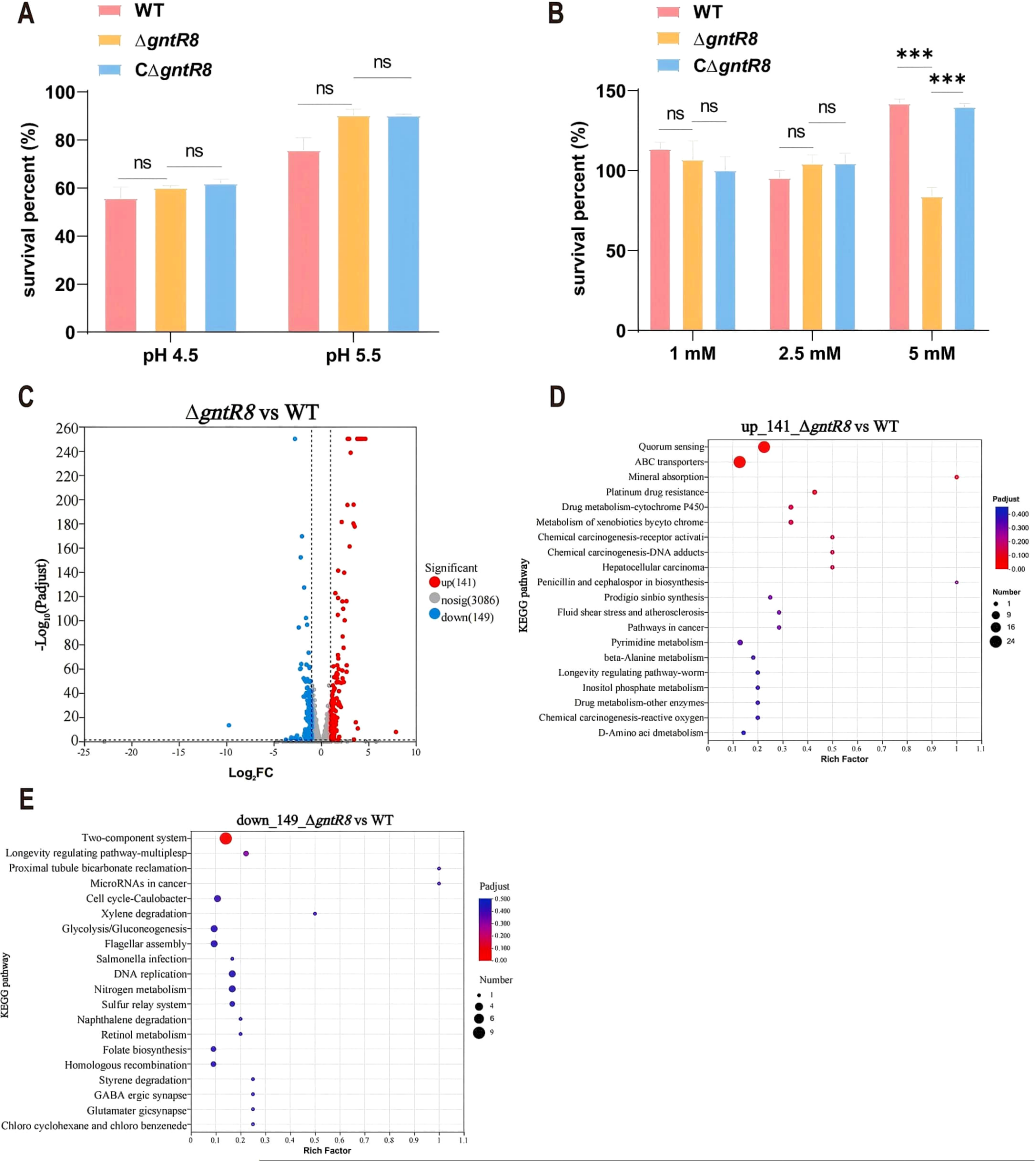
Deletion of the *gntR8* reduces oxidative stress resistance in **(*B*)** *abortus*. **(A)** Survival rate of *Brucella* strains under acidic (pH 4.5 and 5.5) stress. **(B)** Survival rate of *Brucella* strains under H_2_O_2_ (1 mM, 2.5 mM, and 5 mM) at 37°C for 1 (H) **(C)** Treat the counted bacterial suspension with H_2_O_2_ (final concentration 5 mM) for 1 hour. Add RNA protection reagent at twice the volume, vortex, and incubate at room temperature for 5 minutes to extract RNA. Proceed with sequencing processing. Volcano plot of RNA-seq screen for differentially expressed genes. Horizontal coordinates indicate fold difference and vertical coordinates indicate negative Log_10_ values for p-adjust; **(D-E)** KEGG pathway enrichment analysis of up-regulated **(D)** and down-regulated **(E)** genes. Data are presented as the means of normalized data ± standard deviations (error bars) based on three independent experiments. The significance is shown as **p < 0.01; ***p < 0.001; and ns indicates non-significance.

The Dose-response curve showed that the survival rate of the strains gradually decreased with increasing H_2_O_2_ concentration (**Supplementary Figure S3**), compared to WT, the survival rate of ΔgntR8 significantly decreased (p < 0.001) when treated with 5 mM H_2_O_2_. Under acidic conditions, the survival rates of the three strains (WT, Δ*gntR8*, and CΔ*gntR8*) were comparable at pH 4.5 and 5.5 (**Figure 2A**). Oxidative stress experiments showed that, compared to WT, the survival rate of Δ*gntR8* significantly decreased when treated with 5 mM H_2_O_2_ (*p* = 0.0049). In contrast, the survival rate of the CΔ*gntR8* strain recovered to the WT level (**Figure 2B**). RNA-seq analysis was used to identify genes regulated by the *gntR8* gene under oxidative conditions. A total of 290 differentially expressed genes (DEGs) (Fold change ≥ 2 and p-value ≤ 0.05) were identified between the WT and Δ*gntR8*, with 141 genes up-regulated and 149 genes down-regulated (**Figure 2C**). KEGG pathway enrichment analysis sugggested that up-regulated genes may play a variety of biological functions through interaction with quorum sensing system and ABC transporter-related proteins (**Figure 2D**). Down-regulated genes primarily mostly associated with the sulfur relay system and the two-component system (**Figure 2E**). RT-qPCR validated the RNA-seq findings (**Supplementary Figure S4**).

### 3.3 *GntR8* protein specifically binds to promoters of *ALDH*, *gst* and *clpP* genes

**Figure 3:**
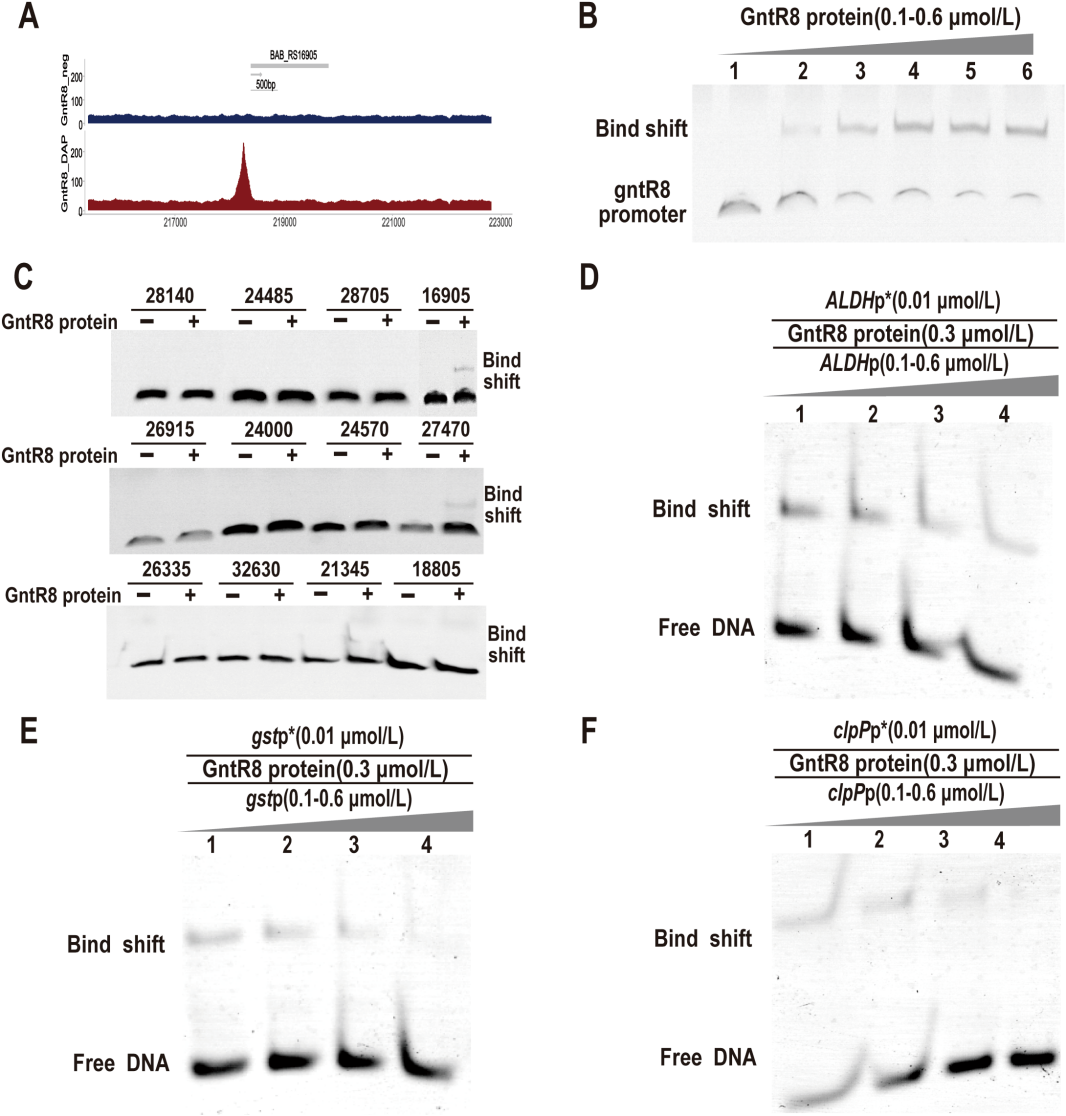
GntR8 protein specifically binds promoters of target genes. **(A)** Dap-seq analysis revealed that GntR8 was enriched in its promoter region; **(B)** EMSA analysis of GntR8 binding to *gntR8* promoter region; **(C)** EMSA assay for GntR8 protein and differential gene promoter DNA sequence; Competition EMSA assays of *ALDH* **(D)**, *gst* **(E)**, and *clpP* **(F)** promoters.

To further analyze the mechanism by which *GntR8* participates in *Brucella* virulence and antioxidant stress, this study utilized Dap-seq technology to analyze the sequences directly bound by *GntR8*, revealing that the binding fragments are all located in the gene promoter regulatory region, potentially indicating self-regulatory functions (**Figure 3A**). Therefore, To analyze whether the GntR8 protein can bind to its own promoter DNA sequence, this study performed co-incubation using the GntR8 protein and the promoter DNA sequence of the *gntR8* gene (BAB_RS24500), and found that the GntR8 protein can bind to its own promoter DNA sequence in a dose-dependent manner, indicating that the EMSA system used in this study can be employed to identify the regulatory genes of the GntR8 transcription factor(**Figure 3B**). Further screening of genes regulated by the GntR8 transcription factor was conducted by amplifying the promoter DNA sequences of potential target genes. The results indicated that the GntR8 protein can bind to the promoter DNA sequences of *ALDH* (BAB_RS16905), *gst* (BAB_RS27470), and *clpP* (BAB_RS21345) (**Figure 3C**). Competitive EMSA assays confirmed specificity, showing progressive inhibition of labeled probe binding upon increasing unlabeled competitor DNA (**Figures 3D-F**). This indicates that GntR8 specifically binds to the *ALDH*, *gst*, and *clpP* gene promoters.

### 3.4 GntR8-mediated regulation of oxidative stress in *B. abortus* via *clpP* gene

**Figure 4:**
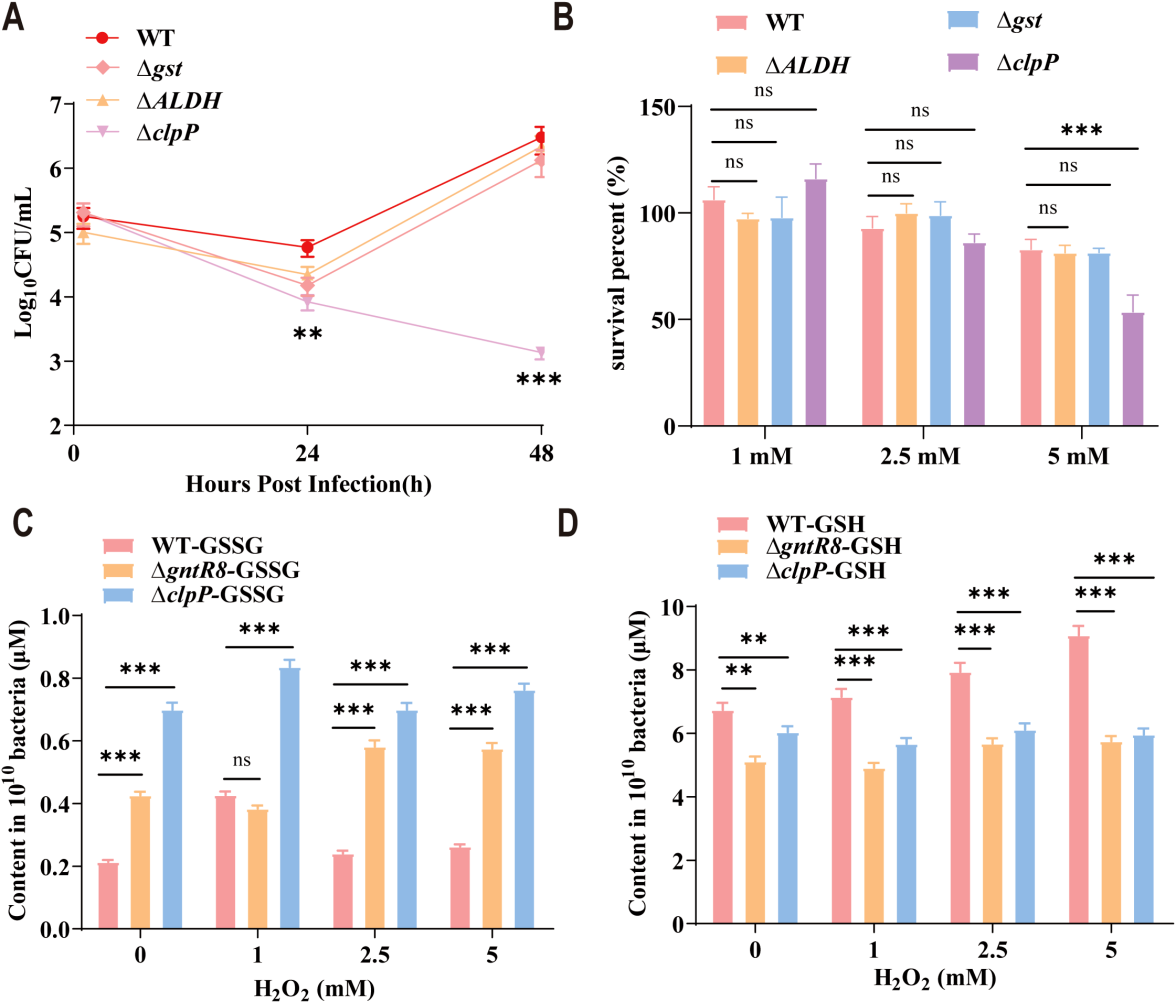
GntR8 transcription factors regulates oxidative stress resistance in *B. abortus* via *clpP* gene. **(A)** Survival rate of WT, ∆*ALDH*, ∆*gst* and ∆*clpP* under stress condition; Total GSSG **(B)** and GSH **(C)** content of *Brucella* strains; **(D)** intracellular survival of the ∆*ALDH*, ∆*gst* and ∆*clpP* deletion strain. Data are presented as the means of normalized data ± standard deviations (error bars) based on three independent experiments. The significance is shown as ** *p* <0.01; *** *p* <0.001; and ns indicates non-significance.

Intracellular survival assay of RAW264.7 cells with Δ*ALDH*, Δ*gst*, Δ*clpP* and WT strains showed that the ability of Δ*clpP* to survive in the RAW264.7 cell was significantly decreased (*p* < 0.001)(**Figure 4A**). Oxidative stress assays with Δ*ALDH*, Δ*gst*, Δ*clpP* and WT strains revealed no significant difference in survival for Δ*ALDH* and Δ*gst* compared to WT. However, after the deletion of *clpP* gene, the antioxidant capacity of *Brucella* decreased significantly (*p* < 0.001) (**Figure 4B**). GSH is an important antioxidant that scavenges free radicals and helps cells maintain normal immune function (37). In this study, the GSSG content of Δ*gntR8* and Δ*clpP* were significantly higher than that of WT (*p* < 0.001) (**Figure 4C**). Consistently, GSH levels were significantly reduced (*p* < 0.001) (**Figure 4D**). The results showed that the antioxidant capacity of *Brucella* decreased after *clpP* gene deletion. These results indicate that GntR8 regulates GSH levels by controlling the expression of the *clpP* gene, thereby modulating the oxidative stress resistance of *B. abortus*.

### 3.5 Deletion of the *gntR8* down-regulates immune-related gene in infected hosts

**Figure 5:**
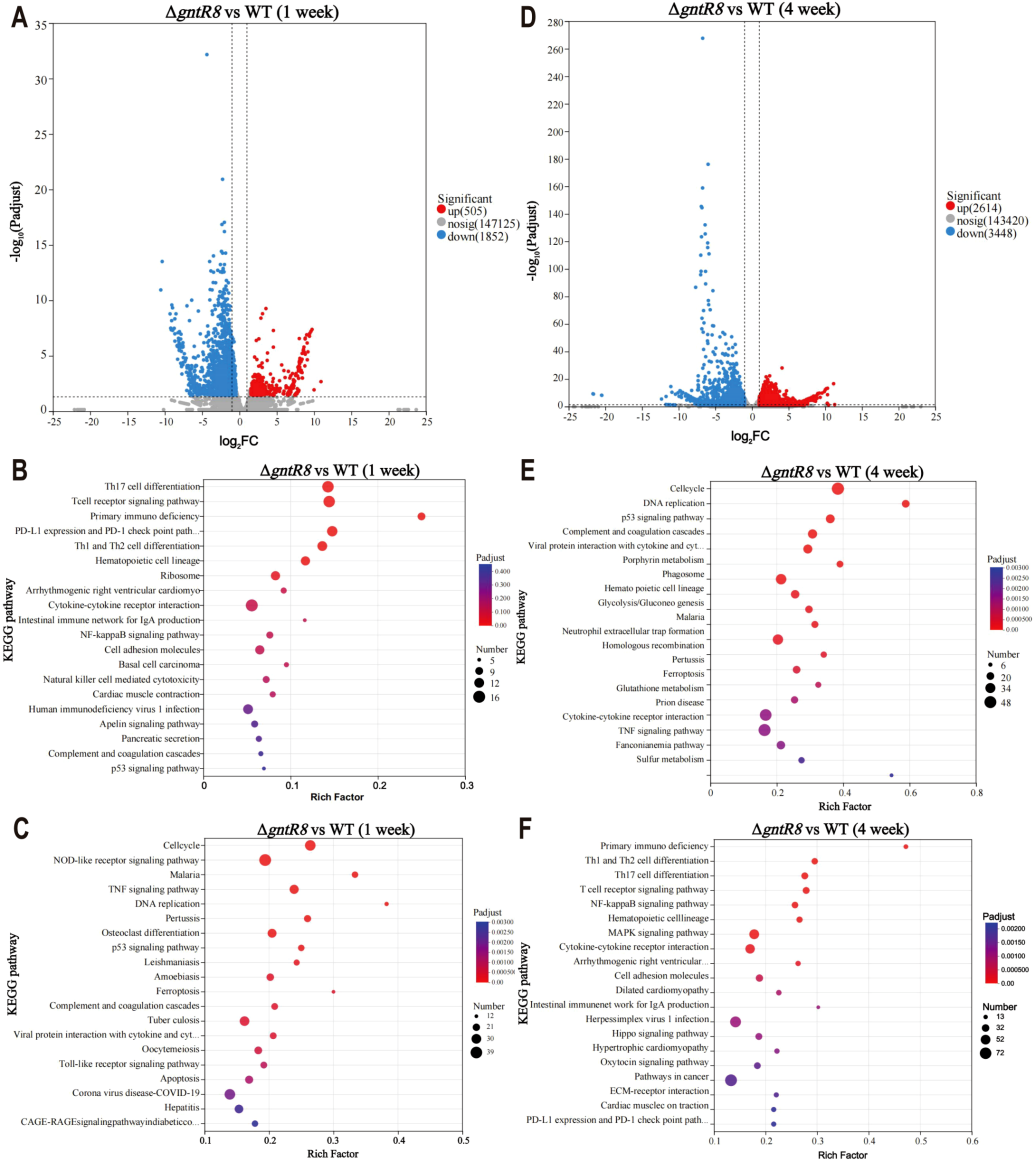
Deletion of the *gntR8* down-regulates immune-related gene in infected hosts. **(A)** DEGs at 1 week post-infection; KEGG pathway enrichment analysis of up-regulated **(B)** and down-regulated **(C)**genes at 1 week post-infection; **(D)** DEGs at 4 weeks post-infection; KEGG pathway enrichment analysis of up-regulated **(E)** and down-regulated **(F)** genes at 4 weeks post-infection. The data is based on experimental results from three mice.

Transcriptome analysis of the spleen of WT and Δ*gntR8-*infected mice revealed that, at week 1 post-infection, 505 genes were up-regulated, while 1852 genes (Fold change ≥ 2 and p-value ≤ 0.05) were down-regulated in the spleens of the Δ*gntR8*-infected mice (**Figure 5A**). KEGG enrichment analysis of up-regulated genes showed that these differential genes were mainly enriched in Th17 cell differentiation and T cell receptor signaling pathway (**Figure 5B**), while down-regulated genes were mainly concentrated in NOD-like receptor signaling pathway and TNF signaling pathway (**Figure 5C**). Similar trends were observed in cell transcriptome results (**Supplementary Figure S5**). At week 4 post-infection, 2614 genes were up-regulated, and 3448 genes were down-regulated in the spleens of Δ*gntR8*-infected mice (**Figure 5D**). KEGG enrichment analysis of up-regulated genes showed that these differential genes were mainly enriched in cell cycle, DNA replication, P53 signaling pathway (**Figure 5E**). Down-regulated genes were mainly concentrated in Primary immunodeficiency, Th1 and Th2 cell differentiation and NF-kappa B signaling pathway (**Figure 5F**). RT-qPCR confirmed RNA-seq results (**Supplementary Figure S6**). Previous studies have shown that immunizing mice with the *B. abortus* 2308 mutant Δ*gntR* can induce classic Th1 and Th2 responses (29). The above analysis showed that the deletion of *gntR8* gene caused the down-regulation of the expression of immune-related genes in infected hosts.

### 3.6 Deletion of *GntR8* reduces cytokine production in *B. abortus*-infected mice

**Figure 6:**
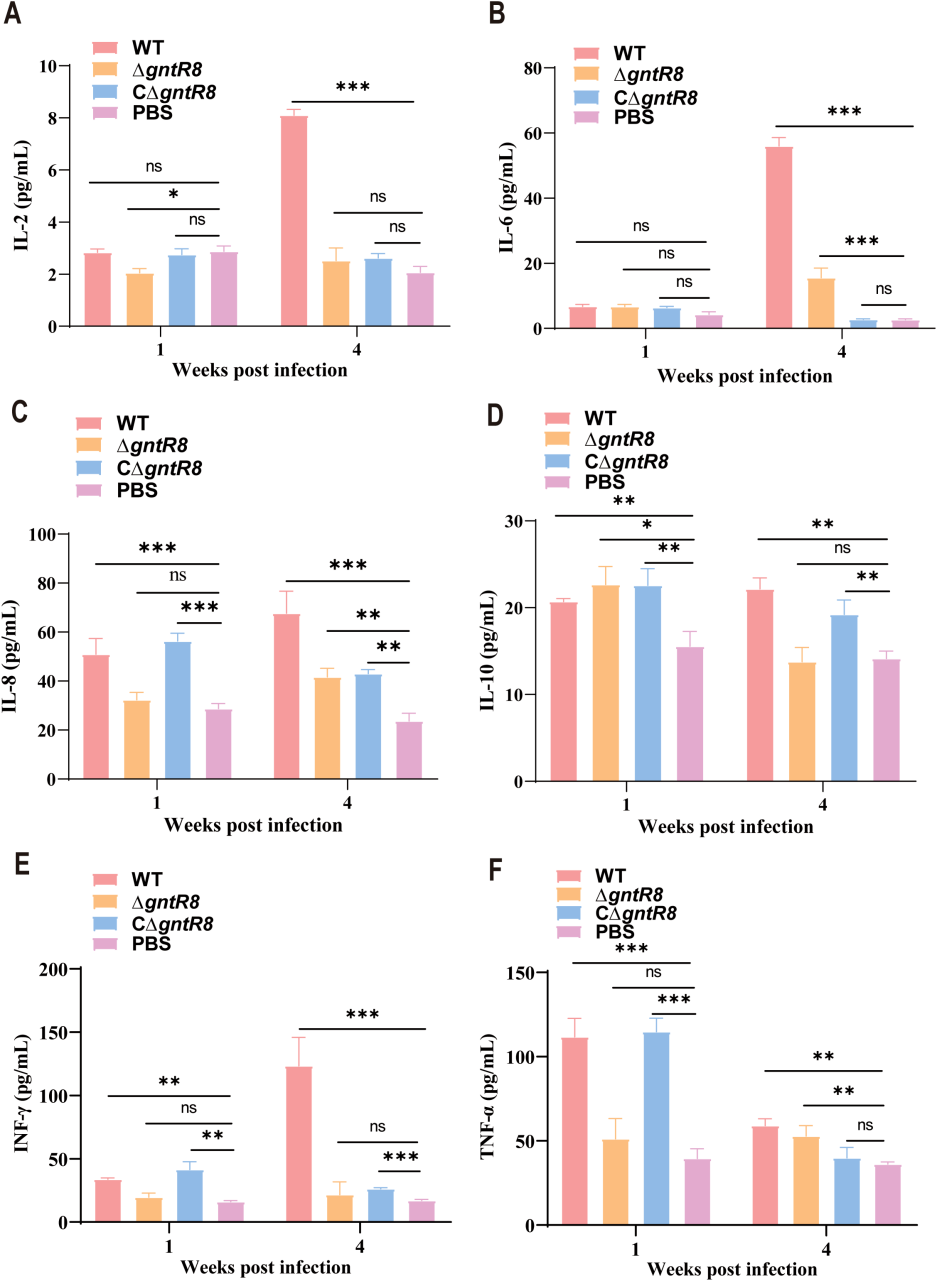
Cytokine production levels post-infection in mice. Production level of **(A)** IL-2, **(B)** IL-6, **(C)** IL-8, **(D)** IL-10, **(E)** IFN-γ, **(F)** TNF-α. Data are presented as the mean ± standard deviation (error bars) of standardized data, based on experimental results from three mice. The significance is shown as *p < 0.05; **p < 0.01; ***p < 0.001; and ns indicates non-significance.

*Brucella*, as intracellular pathogens, mainly relies on cellular immunity for clearance in the early stage of infection (38). Th1 cells participate in the host’s defense against intracellular pathogens by producing IFN-γ, TNF-α, and IL-2, while Th2 cells are responsible for coordinating humoral immunity and participate in the host’s defense against extracellular parasites by secreting IL-4, IL-5, and IL-10 (39–41). Given the RNA-seq findings on immune-related pathways (**Figure 5**), we detected IL-2, IL-6, IL-8, IL-10, IFN-γ, TNF-α and other immune-related cytokines. The results are shown in **Figure 6**, after 1 week, there were no significant differences in cytokine levels between the Δ*gntR8* group and the PBS group. However, compared to the PBS group, the WT group and CΔ*gntR8* group showed significant upregulation of IL-8, IL-10, IFN-γ, and TNF-α. After 4 weeks, compared to the PBS group, all infected groups showed significant upregulation of IL-6, IL-8, and TNF-α expression (*p* < 0.001). This finding indicates that at this time point, Δ*gntR8* induced a Th1-type immune response, consistent with the transcriptomic results. Therefore, we performed additional analyses focusing on MHC-I and MHC-II pathway–related genes. Integrating mouse spleen transcriptome data, we employed real-time quantitative polymerase chain reaction (qRT-PCR) for MHC-I pathway genes (including *H-2K^b^*, *β2m*, *TAP1*, *TAP2*, *Stat1*, and *NLRC5*) and MHC II pathway genes (*CIITA and RFX5*) in mice infected with WT, Δ*gntR8* and CΔ*gntR8* strains at weeks 1 and 4. Results showed that compared to WT, Δ*gntR8*-infected mice exhibited significantly reduced mRNA expression levels for all genes at both time points; CΔ*gntR8* partially or fully restored expression (*TAP1/TAP2* approached WT by week 4) There were no significant changes in the expression of MHC class II-associated genes (*CIITA and RFX5*). Notably, transient elevations of *H-2K^b^* and *Stat1* at week 1 likely reflect early innate/adaptive activation. These results are provided in **Supplementary Figure S7**.

## 4 Discussion

*Brucella* spp., the causative agent of brucellosis, poses a significant threat to both human and animal health (42). Due to its tendency to present clinically as a latent or chronic infection, brucellosis is difficult to diagnose and treat in a timely manner, contributing to its widespread distribution globally (43). Previous studies have highlighted the crucial role of GntR transcription factors in regulating bacterial metabolism and pathogenesis (27). In this study, we demonstrated that deletion of the transcription factor gene *gntR8* in *B. abortus* significantly reduced intracellular survival in RAW264.7 cells and markedly decreased bacterial virulence in mice. However, although RAW264.7 cells possess core macrophage functions (such as phagocytosis and antibacterial activity), as an immortalized cell line, their phenotype may differ from that of macrophages derived from primary monocytes and requires further validation. Splenomegaly and connective tissue proliferation induced by *Brucella* infection in mice represent characteristic pathological features of the intracellular parasitic pathogenesis of this bacterium, as extensively documented in numerous domestic and international studies (31, 44). In this study, pathological analysis revealed that, in mice infected with *B. abortus*, the spleens exhibited varying degrees of enlargement. Histological examination showed connective tissue proliferation in the spleens of WT-infected mice at 4 weeks post-infection, contributing to the spleen enlargement. However, although the complementary strain restored the regulatory pathway required for bacterial colonization by replenishing *gntR8*, it failed to fully reinstate GntR8’s control over “immunopathology-related genes”. This may stem from differences in promoter strength and expression timing between the plasmid vector and the wild-type strain. Consequently, even when bacterial levels reached the target threshold, the spleen’s immune hyperplasia response remained below wild-type levels.

As an intracellular pathogen, *Brucella* spp. invades host macrophages, where it survives and replicates within *Brucella*-containing vesicles (BCV) (45, 46). After phagocytosis by macrophages, the bacteria must adapt to various stressors, including acidic environments, hypoxia, nutrient deprivation, reactive oxygen and nitrogen species (47, 48). Studies on the *Brucella* LysR-type transcription factor BvtR indicate that Δ*BvtR* strains exhibit increased sensitivity to sodium nitroprusside and sodium dodecyl sulfate, but show no altered sensitivity to hydrogen peroxide, isopropyl benzene peroxide, polymyxin B, or natural serum. Deletion of the *OtpR* gene in the *B. melitensis* 16M resulted in reduced tolerance to acidic stress (49, 50). The flagellar transcription regulator FtcR participates in the formation of *B. melitensis* 16M biofilms, which enhance tolerance to hyperosmotic stress (51). *Brucella* enter host cells via interactions between liposomes and macrophage cell membranes, forming *Brucella*-containing vacuoles (BCVs) surrounded by phagocytic vesicles (52). The acidic environment within BCVs facilitates expression of the VirB operon in *Brucella* and regulates T4SS-associated gene expression. *Brucella* utilizes the T4SS to transport effectors from the membrane space into the host cell cytoplasm, thereby modulating host cell signaling pathways to promote its survival within the host (53–55). Thus, *in vitro* models that simulate these stresses are critical for studying the pathogenic mechanism of *B. abortus.* Our findings revealed that deletion of the *gntR8* gene significantly impaired resistance to oxidative stress induced by H_2_O_2_.

Dap-seq is a powerful method used to identify transcription factor binding sites without the need for specific antibody. This technique has been previously utilized in *B. melitensis* to successfully identify the target genes of transcriptional regulators, such as the iron-responsive regulator Irr (56). In our study, integrated Dap-seq and RNA-seq analyses identified 44 potential GntR8-regulated target genes. EMSA further confirmed that GntR8 specifically binds to the promoter of the *clpP* gene, which has previously been implicated in bacterial stress response. *clpP* gene has been confirmed by other studies related functions, the deletion of *clpP* gene can cause *Brucella* to increase the sensitivity to H_2_O_2_, and found that the survival ability of Δ*clpP* in macrophages significantly decreased (31). GSH, a critical intracellular antioxidant, maintains protein thiol groups in reduced states its sulfhydryl moiety. The glutathione peroxidase (GSH-Px)-catalyzed oxidation of GSH to GSSG concomitantly reduces to H_2_O (57, 58). In physiological conditions, reduced GSH constitutes >90% of total cellular glutathione (59). Oxidative stress triggers GSSG accumulation, consequently lowering the GSH/GSSG ratio - a key indicator of cellular redox status maintained through coordinated actions of GSH-Px and glutathione reductase (GR) (60). Our study showed that compared to the WT group, both Δ*gntR8* and Δ*clpP* exhibit compromised H_2_O_2_ tolerance and diminished GSH levels, correlating with impaired intracellular survival. Therefore, it is speculated that GntR8 transcription factor may mediate the antioxidant stress of *B. abortus* through the regulation of *clpP*.

*Brucella* spp. has evolved multiple immune escape mechanisms, with its virulence factors modulating autophagy, inflammation, and apoptosis to suppress the host immune response (61).Transcriptome analysis of *B. melitensis* 16M infected macrophages revealed differential regulation of endoplasmic reticulum-associated pathway, immune-associated pathway, and p53 pathway (62). Notably, infection-induced dysregulation of immune-related genes (e.g., *TXNIP*, *HO-1* and *Prdx5*) has been reported (63, 64), while deletion of the *gntR10* gene elevates levels of TNF-α, IL-6 and IL-8 transcripts in infected cells (65). Our dual transcriptome analyses (host cells and mouse spleen) identified Th17, Th1, and Th2 differentiation pathways as significantly enriched among differentially expressed genes. It has previously been shown that *Brucella* infection triggers innate and adaptive immunity to Th1 and activation of CD8^+^ T cells, reducing MHC-I and MHC-II IFN-γ-induced surface expression and thereby impairing antigen presentation to T cells (66–69). Our cytokine data showed that IL-2 levels were upregulated in the WT group, suggesting that *B. abortus 2308* stimulates specific cellular immunity, whereas deletion of the *gntR8* gene resulted in reduced levels of serum cytokine IL-2, IL-6, IL-10, IFN-γ, and TNF-α production in *Brucella*-infected mice. IFN-γ stimulation rapidly activates the transcription factor STAT1, which subsequently induces IRF1 to upregulate the expression of genes essential for the MHC-I pathway. NLRC5 serves as the primary co-activator for MHC- I genes, playing a critical role not only in their expression but also in maintaining key components of the MHC-I pathway (70–72). H-2K^b^ is a core functional protein in the mouse MHC-I antigen-presentation pathway; its function is to mediate antigen recognition by CD8^+^T cells (73, 74). In Δ*gntR8*-infected mice, reduced interferon-γ levels led to significant downregulation of *H-2K^b^* gene transcription, increased protein degradation, and impaired antigen presentation. This ultimately weakened the adaptive immune capacity for clearing intracellular targets. Concurrently, this study revealed markedly reduced expression levels of *β2m*, *TAP1*, *TAP2*, *Stat1*, and *NLRC5* genes in the spleens of Δ*gntR8*-infected mice. In conclusion, IFN-γ–STAT1–IRF1 signaling and NLRC5 co-activation are attenuated in Δ*gntR8* infection, leading to reduced *H-2K^b^* expression and compromised antigen presentation to CD8^+^T cells.

**Figure 7:**
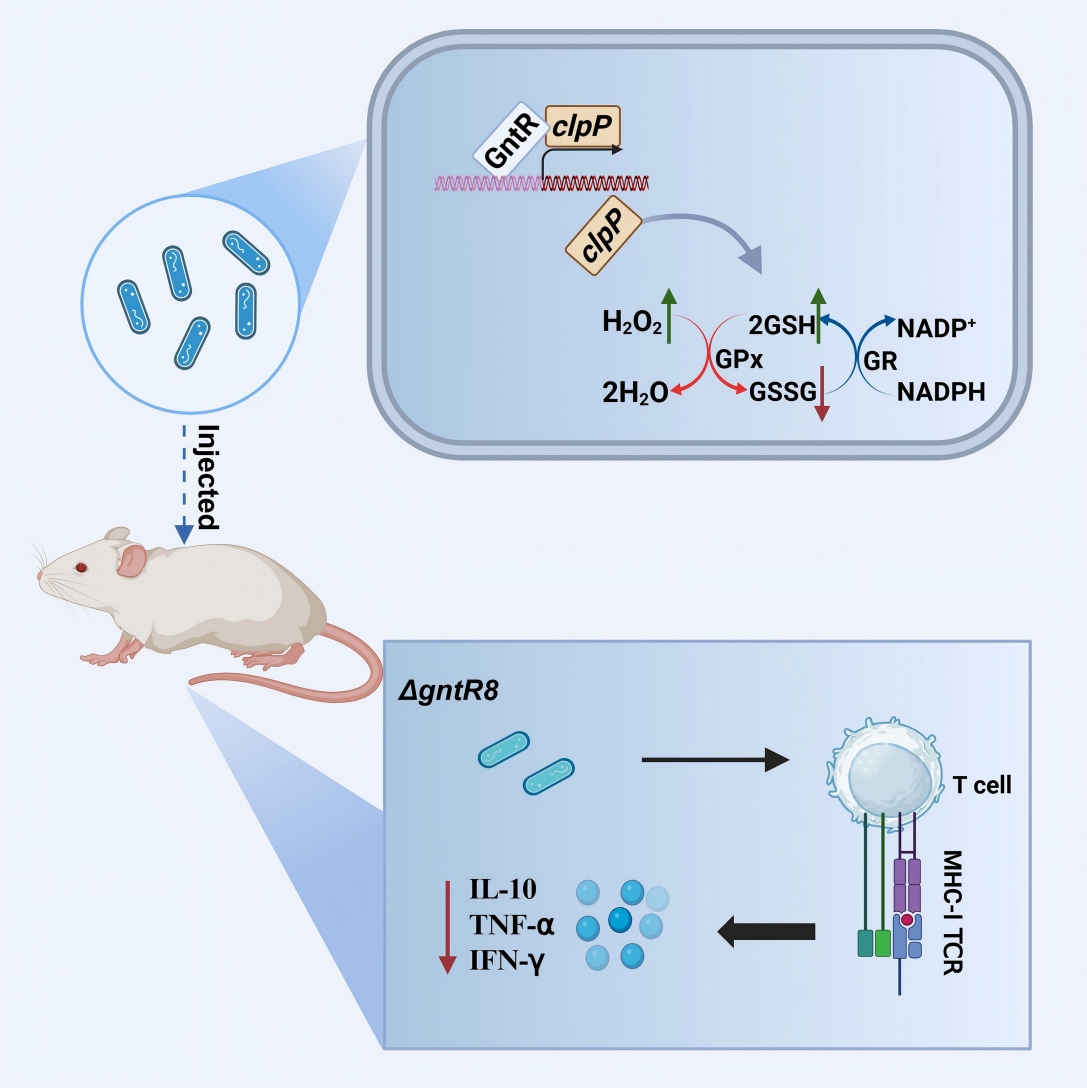
Model summarizing the role of the GntR8 transcription factor in B. abortus virulence. GntR8 directly binds to the clpP promoter, enhancing resistance to oxidative stress, improving intracellular bacterial survival, and potentially modulating host cellular immune responses through MHC-I-dependent pathways. Arrows: Red for decrease; Green for increase; Black for activating effect (Created with bioRender.com)

Collectively, our study establishes the GntR8 transcription factor as a critical regulator of *B. abortus* virulence, intracellular survival, and host immune response modulation. Through combined transcriptomic and binding-site analyses (RNA-seq and Dap-seq), we provide clear evidence that GntR8 directly targets the *clpP* gene, thereby enhancing oxidative stress resistance and intracellular survival. Additionally, we suggest a potential role for GntR8 in immune modulation via MHC-I pathways (**Figure 7**). These findings significantly enhance our understanding of the molecular mechanisms underlying *Brucella* pathogenicity and identify GntR8 as a promising therapeutic target for future strategies aimed at controlling brucellosis.

## Data Availability

The raw Brucella transcriptome data has been submitted to the SRA database with the accession number PRJNA1332959; the raw mouse spleen transcript data has been submitted to the SRA database with the accession number PRJNA1333329; and the raw Brucella Dap-seq data has been uploaded to the SRA database with the accession number PRJNA1333353. All data generated or analyzed during this study are available from the corresponding author upon reasonable request.
