# Supplementary material for: Deletion of the GntR8 transcriptional regulator impairs Brucella abortus intracellular survival and virulence by modulating stress response genes
Source: Front Immunol. 2025 Oct 29;16:1698057. doi: 10.3389/fimmu.2025.1698057 (PMC12604996; doi:10.3389/fimmu.2025.1698057)
Supplement: Supplementary file 2 [file Table2.docx]

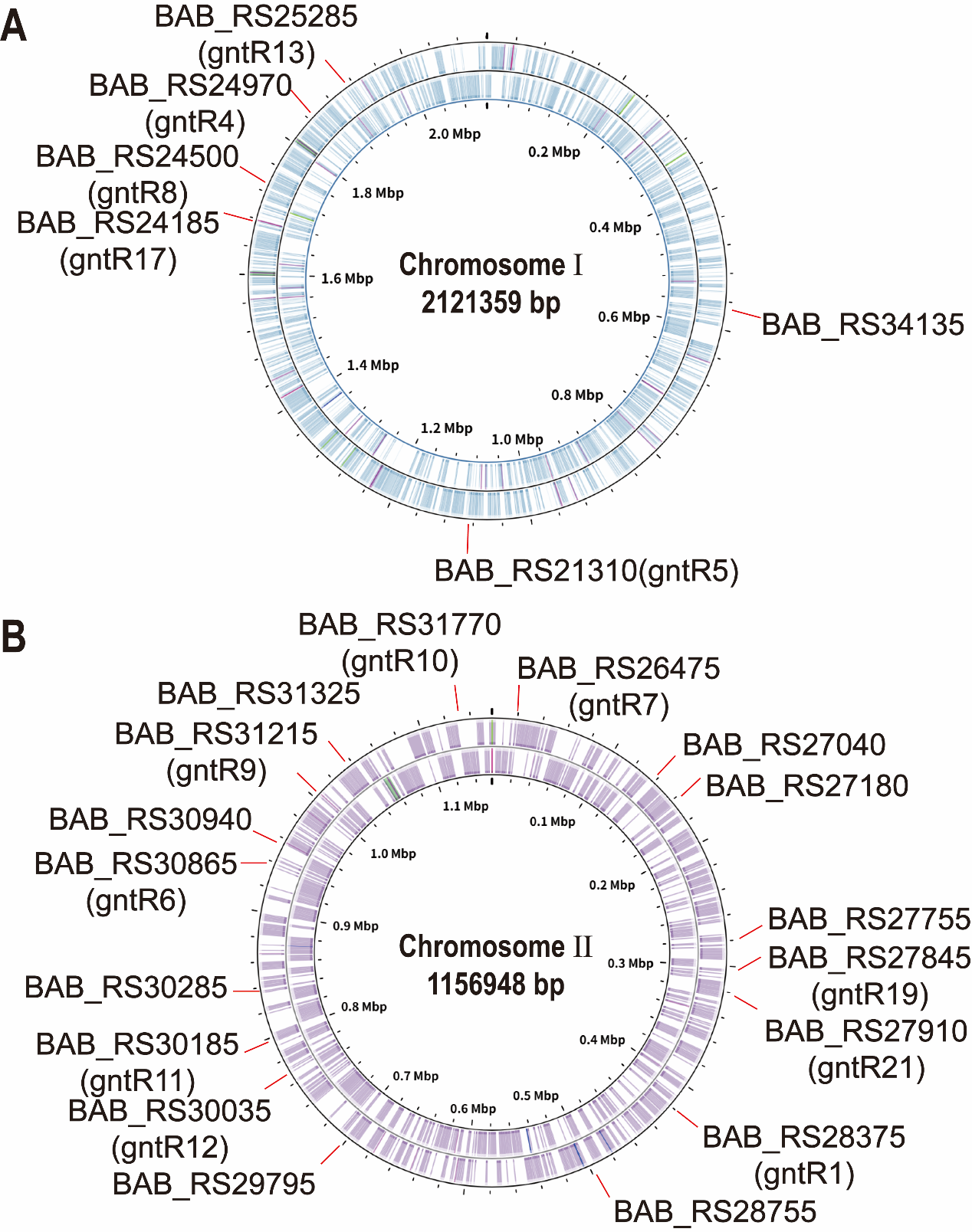


Figure S1. Identification of GntR transcription factors in *B. abortus* 2308 using KEGG and NCBI databases. (A) chromosome I. (B) chromosome II.


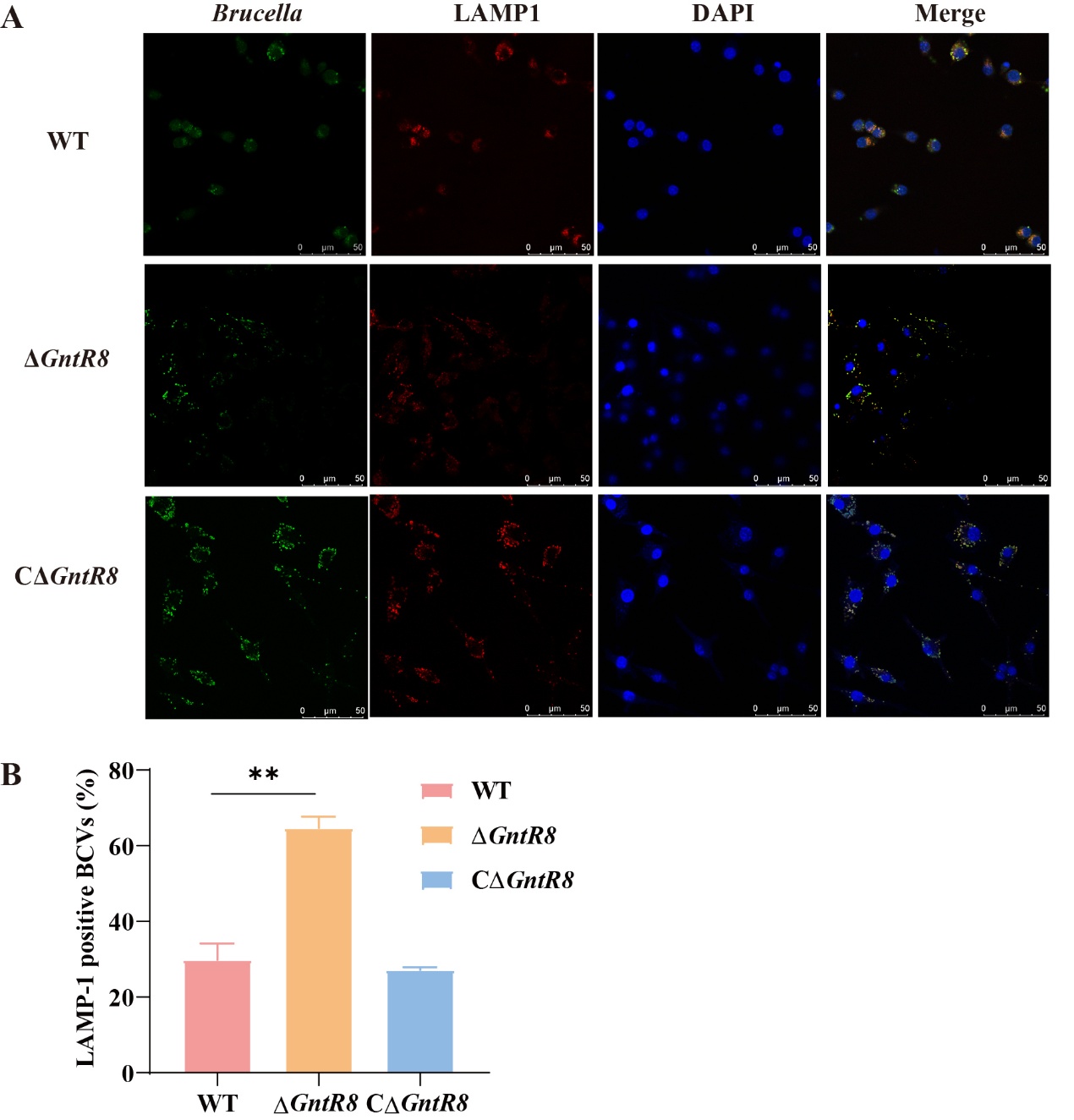


Figure S2: Confocal laser scanning microscopy analysis was determined according to a previous study. (A) RAW264.7 cells were infected with WT, Δ*gntR8*, and CΔ*gntR8* strains, and representative confocal images were captured after 4 hours. *Brucella* are visualized in green, LAMP1 in red, and nuclei in blue. Scale bar: 0-50 μm. (B) The proportion of intracellular *Brucella* colocalizing with LAMP-1 molecules. The significance is shown as ** *p* <0.01; *** *p* <0.001; and ns indicates non-significance.


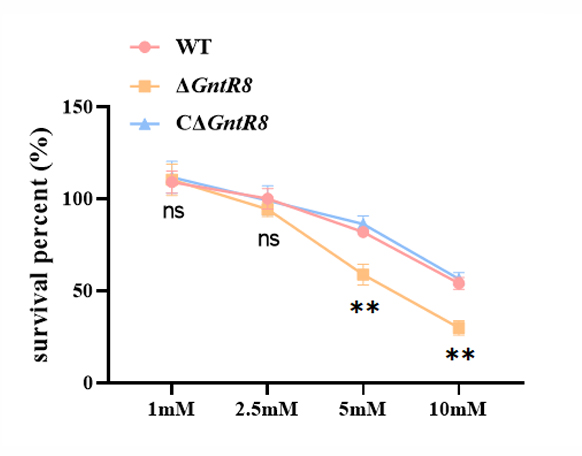


Figure S3: Dose-response curve. WT, ∆*gntR8* and C∆*gntR8* strains were treated with H_2_O_2_ at final concentrations of 1 mM, 2.5 mM, 5 mM and 10mM, respectively. After 1 h of treatment at 37 ℃, surviving bacteria were enumerated by plating serial dilutions on TSA. The significance is shown as** *p* <0.01; *** *p* <0.001; and ns indicates non-significance.


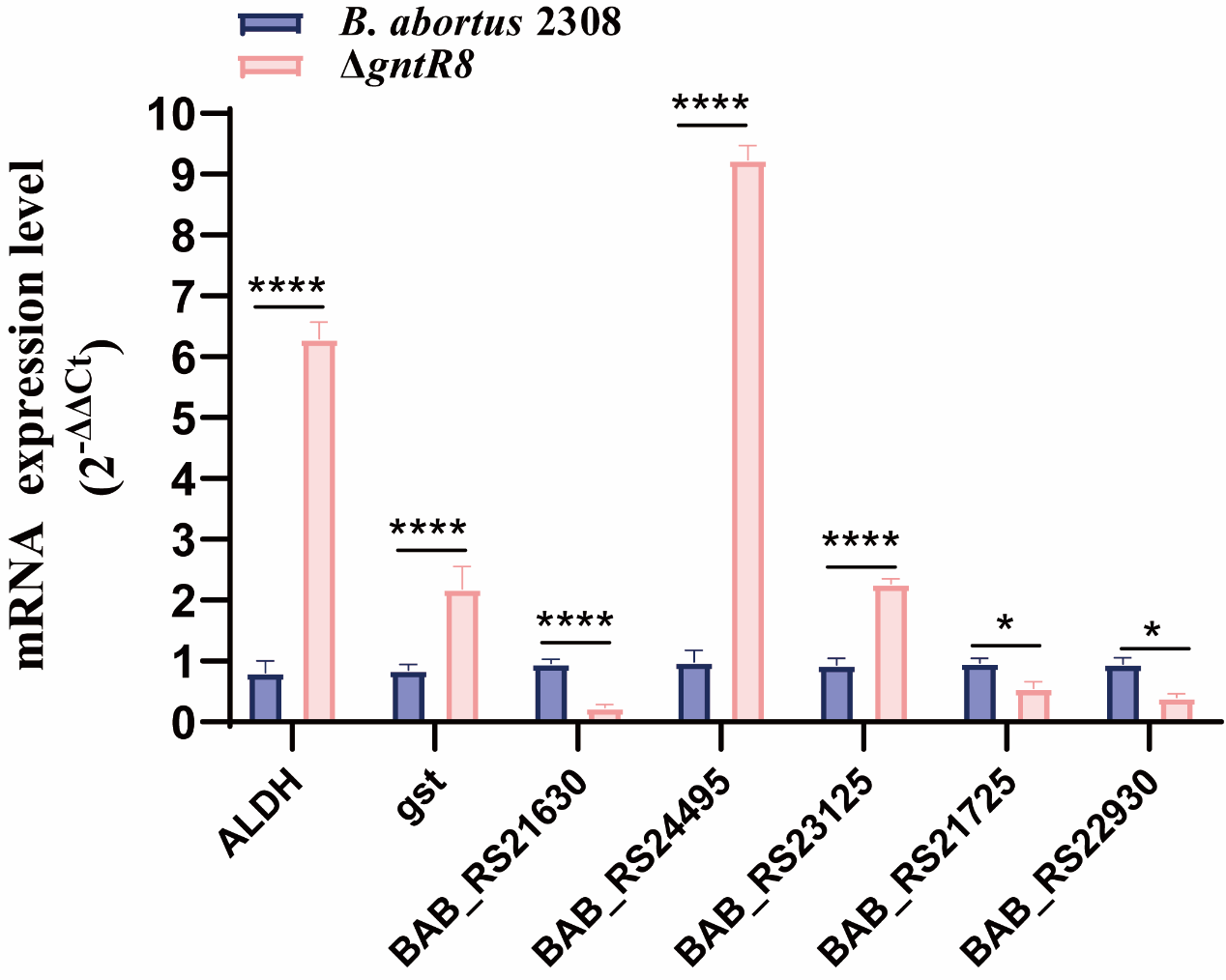


Figure S4. RT-qPCR validation of differentially expressed genes under oxidative stress conditions. Bacterial suspension was mixed with the same volume of H_2_O_2_ (5 mM) and treated at 37℃ for 1 h. Bacteria were collected for RNA extraction, and 16S was used as an internal reference gene for RT-qPCR detection. Data are presented as mean values of normalized results ± standard deviation (indicated by error bars), derived from at least three independent experiments. The significance is shown as* *p* <0.05；** *p* <0.01；*** *p* <0.001；**** *p* <0.0001; and ns indicates non-significance.


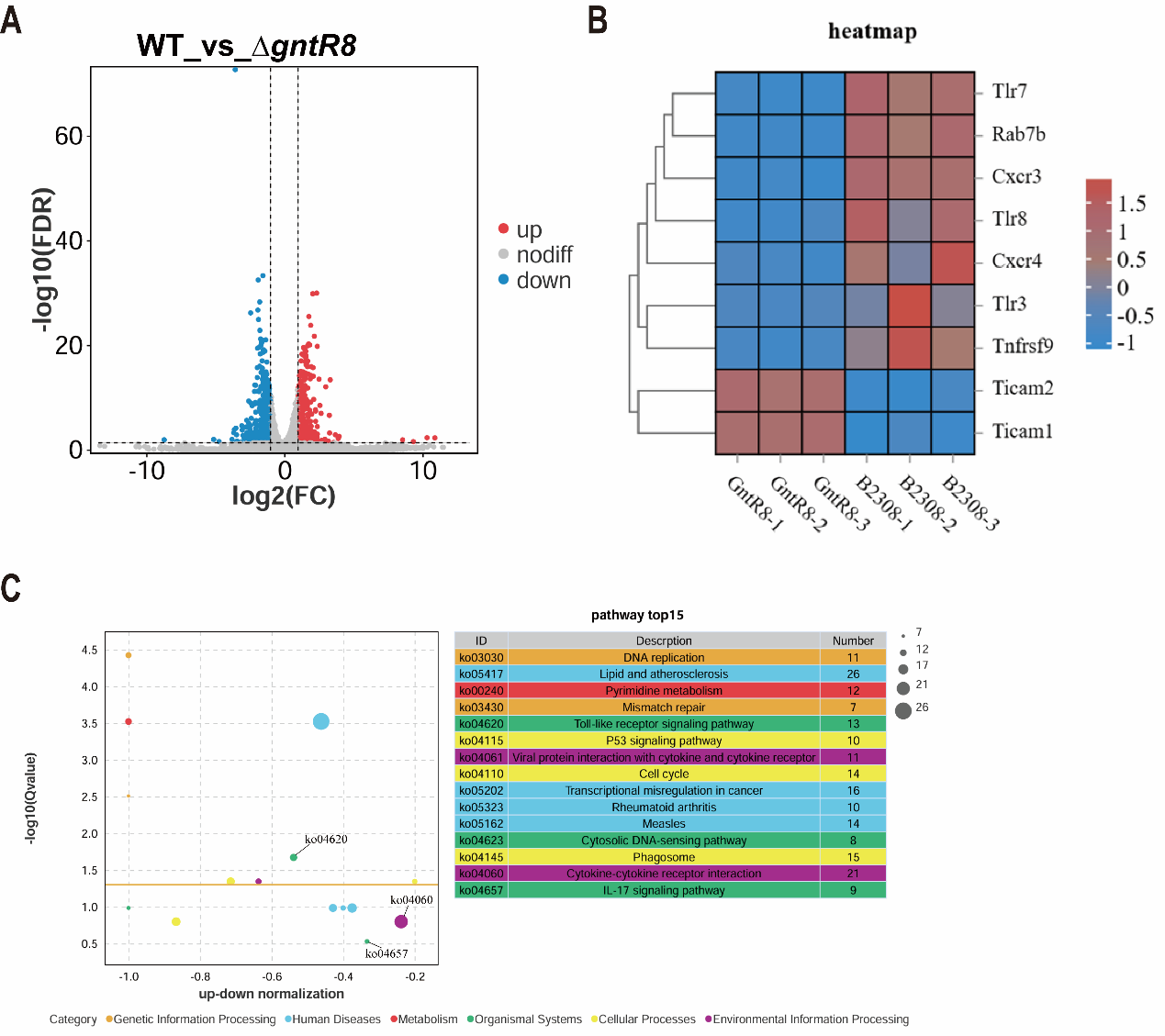


Figure S5. Differential expression of immune-related genes in RAW264.7 cells infected with WT and Δ*gntR8.* (A) Volcano plot of DEGs; (B) Effect of *gntR8* on expression of RAW264.7 cell immune response gene induced by *Brucella*; (C) KEGG pathway analysis. Pathways significantly enriched (FDR < 0.05) are shown above the threshold (yellow line).


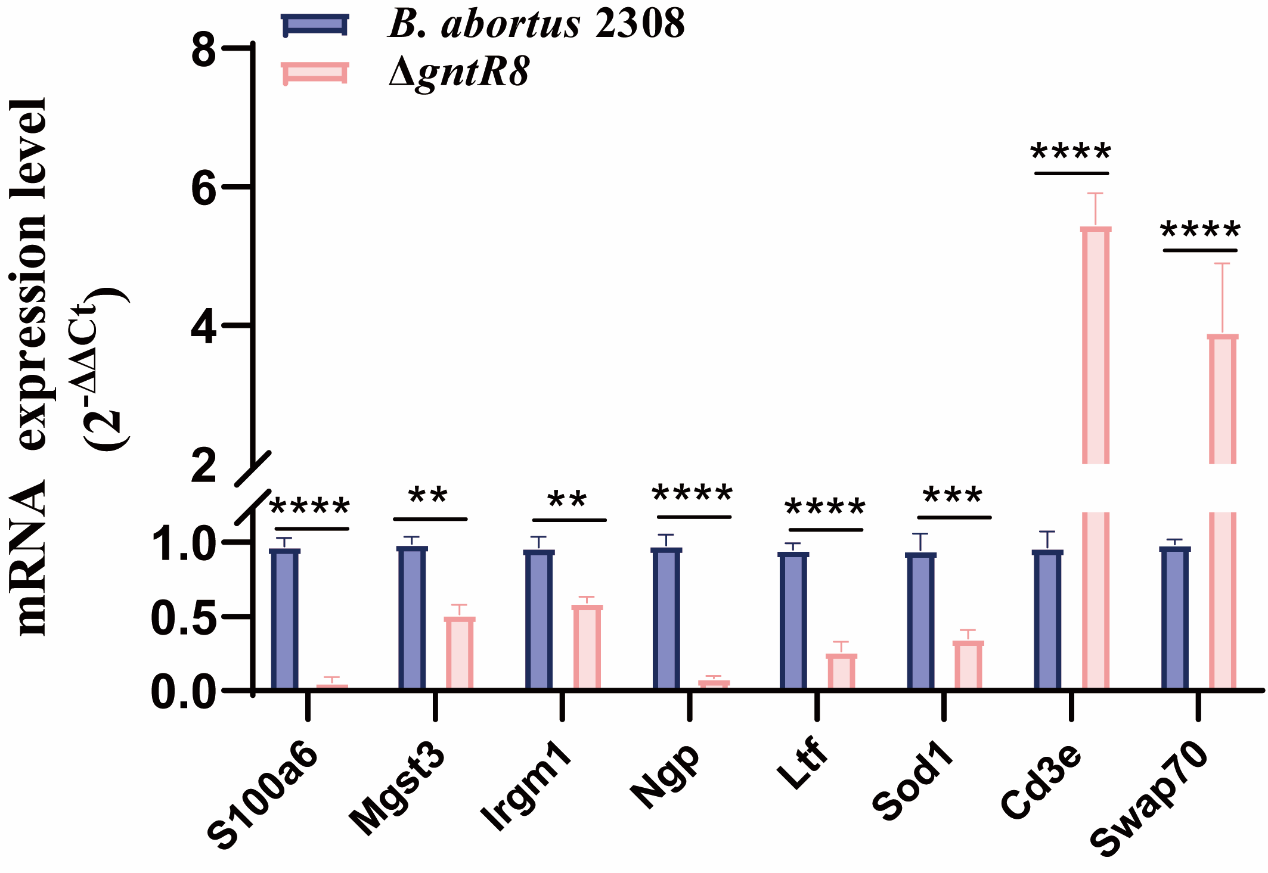


Figure S6. RT-qPCR detection of mouse spleen differential genes. RNA from spleen samples infected with *B. abortus* 2308 and ∆*gntR8* was extracted and subjected to qPCR using GAPDH as an internal reference gene. Data are presented as mean values of normalized results ± standard deviation (indicated by error bars) derived from at least three independent experiments. The significance is shown as * *p* <0.05；** *p* <0.01；*** *p* <0.001；**** *p* <0.0001; and ns indicates non-significance.


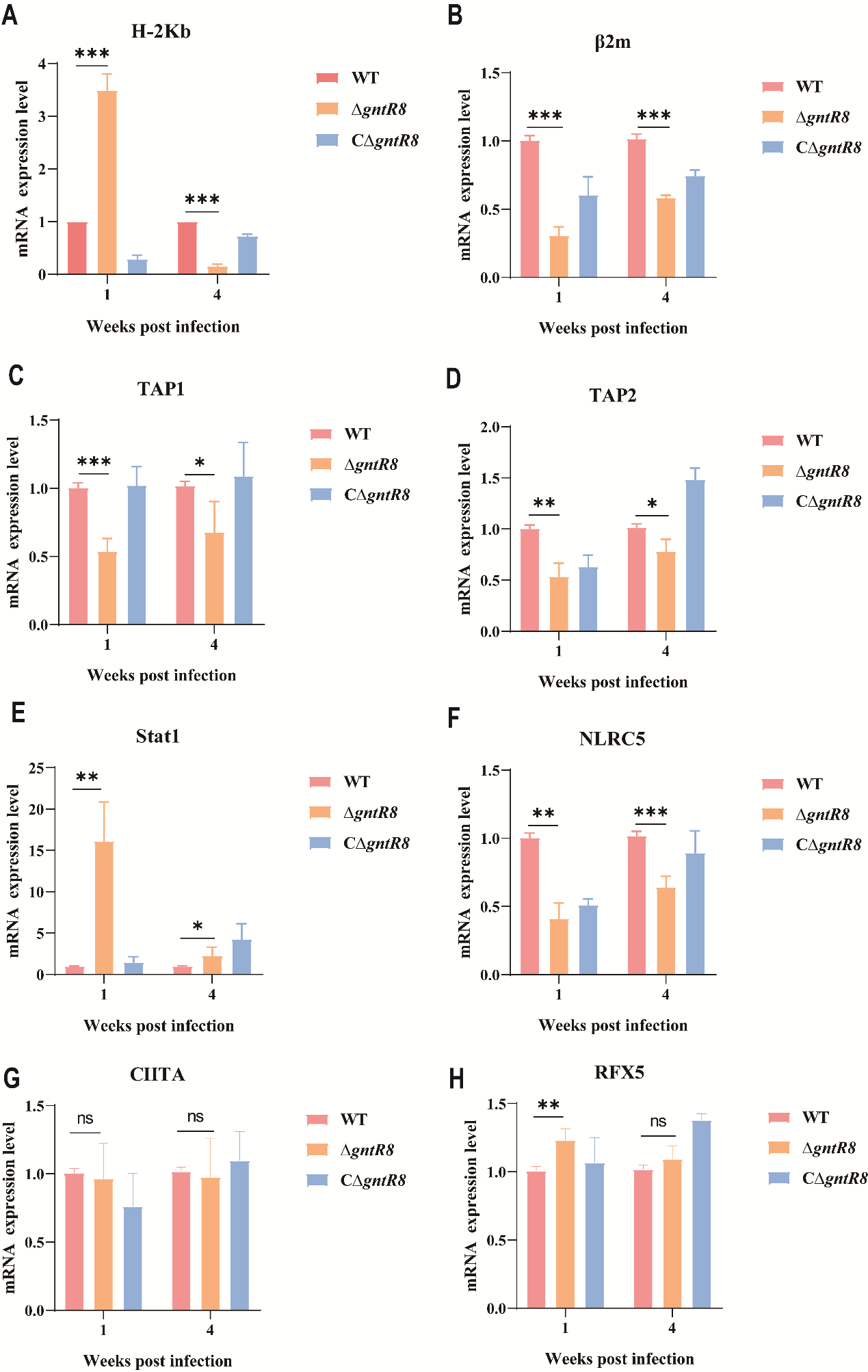


Figure S7: Deletion of the *GntR8* gene reduces the expression of MHC class I-related genes in *B. abortus*-infected mice. Spleen samples were aseptically collected from the mice at weeks 1 and 4, and total RNA was extracted for qPCR analysis. Production level of (A) H-2K^b^, (B) β2m, (C) TAP1, (D) TAP2 (E) Stat1, (F) NLRC5, (G)CIITA, (H)RFX5. Data are presented as the mean ± standard deviation (error bars) of standardized data, based on experimental results from five mice. The significance is shown as* *p* <0.05; ** *p* <0.01; *** *p* <0.001; and ns indicates non-significance.
